# Exploring the Needs of Patients Undergoing Hemodialysis: A Qualitative Study

**DOI:** 10.7759/cureus.50076

**Published:** 2023-12-06

**Authors:** Hayfa Almutary, Reem Al-ghamdi, Zainah Miajan, Amjad Alharbi, Raghdaa Badokhon, Ruba Alharazi, Ohood Felemban

**Affiliations:** 1 Medical-Surgical Nursing Department, King Abdulaziz University, Jeddah, SAU; 2 Public Health Department, King Abdulaziz University, Jeddah, SAU

**Keywords:** patient perspectives, thematic analysis, nurse, need, qualitative study, hemodialysis

## Abstract

Background: Many studies have focused on patients’ experiences living with hemodialysis therapy; however, there is little research exploring their needs. Therefore, the purpose of this study was to explore hemodialysis patients’ needs in Saudi Arabia.

Methods: A qualitative research design with semi-structured interviews was used. Data were collected from Aghrass Medical Center, Jeddah, Saudi Arabia. Data were analyzed using thematic analysis.

Results: A total of 16 hemodialysis patients underwent in-depth interviews. The mean age of the participants was 49 ± 14.93 years of age. More than half of the participants were male (56.25%), and most of them were married (75%). Regarding the clinical characteristics, all patients had three sessions in a week, and the average duration was four hours per session. The mean number of years on dialysis therapy was 3.80 ± 2.8. Using thematic analysis, four themes emerged. These were the impact of fatigue and need for fatigue self-management, need for family and social support, psychological and emotional support from healthcare professionals, and changes in the patients' role performance and their need for adaptation.

Conclusions: This study highlights the aspects of needs among hemodialysis patients from their own perspective. Four themes of needs emerged from this study. Consequently, healthcare professionals should assess patients’ needs frequently to ensure high-quality care.

## Introduction

Chronic kidney disease (CKD) is defined as a persistent impairment of the kidney function. CKD is a global public health problem that is correlated with increased mortality and morbidity [1]. According to epidemiological research, CKD affects between 9% and 13% of people globally [2]. When patients reach the last stage of CKD (stage 5), the kidneys cannot perform their functions sufficiently [1]. Therefore, patients in this stage may require dialysis therapy or transplantation. Hemodialysis therapy is a commonly used form of kidney replacement therapy worldwide among people with end-stage kidney disease (ESKD). In Saudi Arabia, 19,522 patients are currently on hemodialysis therapy for survival out of a total of 28,769 patients [3].

A large body of literature has focused on hemodialysis experience [4-8]. The literature shows that hemodialysis influences different aspects of patients’ lives. These include physical function, psychological well-being, and social and spiritual aspects. Previous studies indicated that hemodialysis therapy has significant impacts on patients’ daily activities, especially on the day of the dialysis session [5,6]. Most patients initiated on hemodialysis suffer from fatigue, which may contribute to an inability to perform their daily activities [9,10]. Another hidden physical stressor is related to sexual dysfunction among hemodialysis patients, which was found to be associated with low quality of life (QOL) [11]. In addition, hemodialysis patients experience several emotional and psychological stressors, such as depression, anxiety, guilt, uncertainty, and fear about the future [10]. According to Al‐Nashri and Almutary (2021), 50% of patients on dialysis had anxiety and 44.7% had depression in Saudi Arabia [12]. In addition, in Saudi Arabia, the percentage of hemodialysis people suffering from depression varied from 5.62% to 44.7% [13]. All these factors may also lead to social isolation and loss of confidence.

A safe environment is another important aspect for patients during hemodialysis sessions. Patients need to feel that their physical environment is safe in regard to concerns, such as vascular access hygiene, and infection control is important for patient safety. Along with the machine alarm for detecting microbubbles that could pass through the tube to the venous line, which decreases the perceived safety of the physical environment, patients feel unsafe when they hear the alarm [14]. A good environment for hemodialysis needs to be open, friendly, intimate, and trustworthy [15]. Although many studies have focused on patients’ experiences living with hemodialysis therapy, there is little research exploring their needs. Patients' needs could differ across cultures. Patients on hemodialysis could have a variety of needs related to physical, psychological, spiritual, and social aspects and could vary across cultures. Nurses should understand patients’ needs from their perspectives in order to provide a high quality of care and improve overall patients’ QOL. Thus, to address the existing knowledge gap, this study was conducted to explore hemodialysis patients’ needs in Saudi Arabia.

## Materials and methods

Study design

A qualitative design was used to explore the needs of hemodialysis patients from their perspectives using semi-structured telephone interviews and a thematic data analysis. This design was deemed the most appropriate approach for the current study because it explores experiences and phenomena from the point of view of the involved people when little is known about the investigated topic.

Participants

Data were collected from Aghrass Medical Center, Jeddah, Saudi Arabia, for two months between May and June 2020. A purposive sampling method was utilized to select participants. The study recruited eligible participants from the dialysis unit. Participants were eligible to participate if they were adults 18 years old or older, had undergone hemodialysis therapy for at least six months, were able to understand and speak Arabic, and were willing to participate. The exclusion criteria were patients on peritoneal dialysis or with kidney transplantation and those who needed a special care, such as hemodynamically stable patients or those with cognitive impairments. All eligible participants were invited through a call to participate in this qualitative study. Sampling was conducted until data saturation.

Data collection

After ethical approval for the study was obtained, the nurses in charge at the dialysis unit were contacted to help recruit eligible participants for this study. As this study was conducted during the quarantine period due to the COVID-19 pandemic, the contact number of all eligible participants was taken with the assistance of the head nurse. The participants were randomly contacted by phone to ask for their willingness to take part in this study. One researcher contacted each participant to arrange for a suitable interview time.

Before the beginning of the interview, the purpose of the research was explained verbally to each participant. In addition, consent to participate and to recording were obtained verbally at the beginning of the interview. The interviews were conducted by two researchers who were trained to conduct this interview and were aware of the objectives of the study. The participants were divided randomly among them, so each one had a full interview. This helped reduce disturbances during the interview and made the participants more comfortable. The participants talked about their needs in a varied and informal manner. Answers were recorded by note-taking and call recording with participant consent.

Telephone interviews were conducted using a semi-structured interview guide to explore the needs of patients undergoing hemodialysis in depth. The interview guide was developed by the research team to ensure feasibility of the study and allow participants to express their feelings and needs. A semi-structured interview guide was created according to the most recent research in this topic and the researchers' prior experiences. All participants answered a set of broad open questions that were prepared by the research team. These questions asked about participants’ perceptions of their physical, psychological, and social needs and finally how hemodialysis has changed their lives. Table 1 presents the semi-structured interview guide.

**Table 1:** Semi-structured interview guide

Semi-structured interview guide
What are your perceptions of your current physical needs?
Can you describe the physical needs that are important to you?
What are the psychological and emotional changes you have faced since starting hemodialysis?
What is your perception of your current psychological needs?
Is there anything else that you want to share about your psychological needs?
How did hemodialysis affect your family and social role? And from your point of view, what social needs do you have?

In addition, the interview began by obtaining the demographic characteristics of all participants (i.e., age, marital status, level of education, date of onset of kidney disease, frequency, and duration of hemodialysis sessions). Then, the participants were asked to express and explain their experiences with CKD and their needs (physical, psychological, and social). The participants were interviewed once, and the duration varied between 30 and 40 minutes.

Data analysis

All data were analyzed using thematic analysis, guided by Braun and Clarke's thematic analysis method [16]. The analysis started when the data were collected. The first step was to become familiar with the data by transcribing them verbatim and reading and re-reading the data, together with discussing refinements of the interview guide. After that, initial codes describing the data were generated in a systematic manner. Subsequently, similar codes were grouped to form potential themes. Afterward, these potential themes were reviewed in relation to codes. The analysis continued by naming and explaining (i.e., developing a clear definition) each theme. The last step in the analysis was writing the results based on the research questions and literature. All the analysis processes were performed manually by the research team. Data saturation was reached after the 14th interview. However, the data were complemented with two additional interviews to confirm the potential themes and ensure that the sample had maximum variation.

Study rigor

The rigor of this study was ensured according to Lincoln and Guba's criteria. Therefore, different strategies were applied to ensure the accuracy and credibility of the research results. The peer-checking process was applied to ensure the credibility and trustworthiness of the findings. In addition, reviewing the audio recording and audit trail of interviews enhanced the confirmability of the findings. Moreover, the research team conducted several analytic meetings to avoid bias or dominating a single perspective, which confirmed the reliability of the findings. Lastly, transparency was guaranteed by precisely outlining and recording the analysis procedure.

Ethical considerations

Approval was sought by the Institutional Review Board Committee at Faculty of Nursing, King Abdulaziz University, Jeddah, Saudi Arabia (reference no. 471-19). Verbal informed consent was obtained from the participants. The participants were provided information about the purpose of the study, methods, and procedures. Moreover, they were informed about their rights to voluntary participation and withdrawal at any stage of the study without any negative consequences for their treatment or care.

The interviews were recorded after permission to tape-record the interview was obtained from the participants. The anonymity and confidentiality of the data were preserved during the study through multiple methods. The audio recordings were transcribed and anonymized, and the participants were provided certain code numbers instead of their identified names to use in the information sheet and interview transcripts. Second, data were kept on an encrypted password-protected laptop, and only the researcher team had access to them.

## Results

This section presents the findings from the analysis of 16 hemodialysis patient interviews. These are the impact of fatigue and need for fatigue self-management, need for family and social support, psychological and emotional support from healthcare professionals, and changes in the patients' role performance and their need for adaptation. The results are presented in two sections. The first section presents the sample characteristics, and the second section presents the main findings.

Demographic and clinical characteristics of the participants

Table 2 provides descriptive statistics to summarize the sample characteristics. The mean age of the participants was 49 ± 14.93 years of age, ranging from 23 to 68. More than half of the participants were male (56.25). Most of the patients were married (75%), and all lived in Jeddah City. Regarding the clinical characteristics, all patients had three sessions in a week, and the average duration was four hours per session. The mean number of years on dialysis therapy was 3.80 ± 2.8, which ranged from 1 to 10.

**Table 2:** Descriptive statistics to summarize the sample characteristics (N = 16)

Variable	M (SD)	n (%)
Age	49 ± 14.93	
Gender		
Male		9 (56.25)
· Female		7 (43.75)
Nationality ·		
Saudi		6 (37.5)
Non-Saudi		10 (62.5)
Marital Status		
Single		3 (18.75)
Divorced		1 (6.25)
Married		12 (75)
Educational level		
Low (≤ higher secondary school)		6 (37.5)
High (> higher secondary school)		10 (62.5)
Years of dialysis	3.80 ± 2.8	

Main findings

Four themes emerged about the aspects of needs for hemodialysis patients. These included the impact of fatigue and need for fatigue self-management, need for family and social support, psychological and emotional support from healthcare professionals, and changes in the patient’s role performance and their need for adaptation. Table 3 summarizes these themes with sub-themes and quotations.

**Table 3:** Summary of themes with sub-themes and quotations for physical, psychological, and social needs.

Most patients indicated that the fatigue they experienced during and after hemodialysis sessions was a main problem they commonly faced, and they felt that this issue needs more attention. Fatigue levels differed from one patient to another, but it affected their daily activity and QOL. The participants emphasized that their need for rest and sleep after dialysis sessions. The participants also described that physical fatigue has a significant impact on family roles and relationships.		
Theme	Sub-theme	Supporting quotation
The impact of fatigue and need for self-management of fatigue	Need rest to restore energy	“After sessions, I need three to four hours of rest because I suffer from muscle tension in my hand and leg and feel fatigued.” (participant no. 4)
	Need sleep due to fatigue that is secondary to a long dialysis session	“…after the hemodialysis sessions, I have fatigue, dizziness and I don’t feel like talking to anyone. So, I need to sleep because the 4 hours (of the dialysis session) takes a long time.” (participant no. 3)
	Need time to restoring energy after dialysis session	“After the hemodialysis session, I want to rest for 1-2 hours to restore my energy so I can do my housework and take care of my kids.” (participant no. 2)
	Need rest and sleep after the dialysis session	“After the session, I barely rest and sleep for an hour, then when I wake up, I feel very hungry. I can't move; my body feels weak to the point that I can't even pray.” (participant no. 4)
	Need sleep after dialysis session, need to get symptoms managed after dialysis	“Usually, when I go back home after dialysis, I sleep immediately; I can't raise my head or even my hands. I wake up the second day and I feel so dizzy and tired, and I can't stand up well, my legs hurt me ... I mean very weak; I can't stand up for a long time ... it is really a big problem. I need treatment for this.” (participant no. 8)
	The duration of fatigue is all-time, which constricts people.	“I am always in bed or on the chair. I feel tired all the time.” (participant no. 9)
	Tiredness varies from session to session, which deteriorate a person	“Sometimes I feel good after dialysis, but other times I feel very tired and need a full rest. I think I am deteriorating with time.” (participant no. 15)
	Fatigue affects performance responsibilities.	“When I return home, I feel tired, I don’t have anyone to take care of my children, and my husband is always working. I feel tired and if it wasn’t necessary, I wouldn’t take care of them. I don’t want responsibilities.” (participant no. 2)
	The fatigue hinders doing daily activities.	“Before hemodialysis, I was running everything in the house; now, I can't. Some days when I have a little energy, I do simple things like folding clothes, washing a few dishes.” (participant no. 5)
	Tiredness affects the ability to enjoy things.	“I cannot enjoy things as I did before. Most of the time, I am tired, especially after dialysis.” (participant no. 11)
	Feeling grief due to fatigue restraints	“Sometimes, when I see my children need something and I cannot provide for them because I cannot work long hours because of the fatigue that I feel due to dialysis, I grieve for myself and get tired of myself, but we say, thank God for every situation.” (participant no. 7)
Some of the dialysis patients explained that they need psychological, and family supports them, which is very important during the treatment period and helps to improve their psychological condition and feel comfortable accepting treatment. Also, the participants emphasized their need for psychological, family, and community support during hemodialysis treatment.		
Need for family and social support	Need family support to express feelings	“I need my family support after the session, like rest, complimenting me and asking how I feel; I need to feel that I’m not a burden to them in all aspects.” (participant no. 2)
	Need social support	“I need the people around me to understand me and to help me.” (participant no. 6)
	Need family and social support to express feelings	“I need support from my family, husband and community ... I need my husband to understand me, I need him to talk with the doctor so he can understand me.” (participant no. 2)
	Feel upset and need family support	“My family is far away from me, and the dialysis sessions started on my own without any support. All of this make me feel upset … but I am still thankful to God.” (participant no. 3)
	Family support relieves psychological stress.	“It would be better if I had my family here with me. Because when I talk to my wife, I forget all my sadness and I feel comfortable. If I can’t talk to her, I am really upset. For that, I really need them here with me.” (participant no. 3)
	Psychological and emotional support from family and friends are essential.	“Without psychological and emotional support from family and friends, it is impossible to continue the treatment.” (participant no. 14)
	Social support is essential.	“It would be a hard life without those supportive people around us. No one can survive alone without support. I guess that.” (participant no. 11)
The participants emphasized that they needed more understanding and support from nurses and healthcare providers.		
Psychological and emotional support from healthcare professionals	Need reassurance from healthcare professionals.	“I need to feel that I’m not a burden to the doctors.” (participant no. 2)
	Nurses need to listen to repeated complaints.	“I need nurses to listen to me ... even when I have the same complaint each time.” (participant no. 9)
	Need to express feelings to healthcare professionals	“When I get so exhausted from dialysis sessions, I feel that I need a doctor or psychotherapist to talk to. From time to time, I become down.” (participant no. 16)
	The need to express feelings to a psychologist	“The stressors are the most important issue. I have a lot of stressors, such as family issues, housework and taking care of my kids. Going to a psychiatrist once a month is good to express my feelings.” (participant no. 2)
	Psychological support after dialysis session is needed.	“The important thing is psychological comfort. I need to find comfort when I am back home from my dialysis session. I have kids to take care of and responsibilities.” (participant no. 2)
	Need support when sad	“Some situations make me sad. I feel I need support. Like when I propose to a girl, her family refuses because I am on hemodialysis.” (participant no. 1)
	Need to hear supportive and kind words	“Sometimes, when you complain and someone tells you that God will help you, or even hearing kind words, it makes you feel better and calm.” (participant no. 5)
	Need reassurance	“Each time that I am connected to the machine, I feel that I might die or get a serious infection at any moment ... please tell me that wouldn't happen” (participant no. 13)
	Need reassurance	“When I have a check-up, I always feel scared of the results. I am afraid that my condition is getting worse ... I heard that some people get heart problems after a while from dialysis. What could I do to relax?” (participant no. 12)
Some patients reported that they faced changes in their ability to normally function on hemodialysis. This caused them to need assistance from their families. These changes had an effect on their role as mothers and fathers, as is clear in the following quotations:		
Changes in the patient’s role performance and their need for adaptation	Unable to work	“By God, I cannot work like before. After I started dialysis, my wife, and my son, they became responsible for spending on the house and taking care of my children and my little girls.” (participant no. 7)
	Need assistance from family to do daily responsibilities	“I had to bring my son from Sudan in order to take responsibility for the house and to drive his sisters to school, because I couldn't maintain the routine after I started hemodialysis.” (participant no. 4)
	The patient and the family need to adapt to changes after dialysis.	“I need my husband’s support and understanding for me doing my role completely, which is exhausting for me as a hemodialysis patient. It’s not just me who needed to adapt to these changes; my family has also had to change.” (participant no. 2)
	Unable to work and feel burden on family.	“I cannot find work and my family pays my expenses; I am a burden for them. It is supposed that I am an adult and do something for them.” (participant no. 15)
	Need to adapt to changes after dialysis	“I can’t function like before. Before the dialysis, I was able to play, travel and hang out. But now, I’m just sitting in the same city … I need to accept this situation.” (participant no. 1)
	Feel burden on family	“When I just started hemodialysis, two of my sons had to support me from each side and walk me like a two-year-old kid ... I feel like I am a burden to them.” (participant no. 5)
	Need adaptation	"In fact, despite the long period of time that I have been on dialysis, I still need to adapt more to the situation of my inability to perform all my roles as a mother and housewife." (participant no. 10)
	A change in family roles should be accepted.	“It is not easy to accept that your family members carry out your tasks because of dialysis, especially since I do not want anyone to see my need or weakness. But reality requires me to accept this situation.” (participant no. 16)

## Discussion

This qualitative study explored and offered an enriched understanding of hemodialysis patients’ needs from their own perspectives in Saudi Arabia. Living with hemodialysis treatment creates many stressors and restrictions in patients’ everyday lives. Four main needs for hemodialysis patients emerged. This study found that most participants experienced fatigue during and after hemodialysis sessions, which had an impact on their ability to perform daily life activities. This result is well documented in the literature, as fatigue is reported as the most prevalent symptom among patients receiving hemodialysis therapy [9,17]. Several factors could contribute to a lack of energy and increased fatigue levels among patients receiving hemodialysis therapy. The occurrence of other symptoms, such as pain and sleep disturbance, could increase fatigue [18]. A previous study also showed a correlation between hemoglobin level and functional capacity [10]. Dialysis duration may also contribute to an increased level of fatigue and the need for rest, which can affect QOL. Several studies have observed that dialysis duration has a negative impact on QOL [10,14]. Fatigue has a significant negative impact on the performance of daily activities and consequently QOL [5,10]. Patients suffering from fatigue may have special needs distinct from others, and distress could be associated with the severity of fatigue levels, which may warrant further research.

Many patients in this study indicated that they need rest and sleep after the hemodialysis session. Sleep disturbance is a common problem among patients undergoing hemodialysis [19] due to uraemia-related factors [20]. Studies have also found associations between sleep disturbance and fatigue among patients with CKD [21,22]. Evidence suggests that the quality of sleep and rest is significantly improved on the day of dialysis [23]. Therefore, patients found this to be a necessary need to help them conserve their energy to maintain their daily routine and fulfil their family roles and relationships [24]. Nurses may need to encourage patients to make a schedule for sleep and rest that includes hours of rest and comfort after the dialysis session, according to the patient’s condition.

We also found, through interviews and analysis of results, that many dialysis patients need to be in a safe environment and supported from family, doctors, or nurses, as it provides them with psychological comfort and helps them to continue and adhere to treatment. This finding is consistent with a previous study, which suggests that a safe environment is one of the most important priorities for patients during hemodialysis sessions. A similar study has been conducted in Saudi Arabia exploring psychological needs from different perspectives [14]. Furthermore, in our study, communication between healthcare providers and families with dialysis patients was found to be extremely important from the patients’ perspective, as it provides them with more emotional support and noticeable improvement in their health. While the family of patients and health care professionals can provide psychological support, it is preferable to assess patients’ psychological condition periodically using validated measures for early dedication of any cases that require early management and support. Nurses may also refer patients who need family support to social workers, who may suggest a schedule for the family to assist patients in their daily activity in a manner that suits their health condition to improve their quality of life.

Another important issue indicated in this study was related to patients experiencing changes in their roles and the ability to function normally as before hemodialysis. A previous study found that hemodialysis greatly limits physical activity and affects patients' QOL even when they do not experience multiple complications [14]. Several factors may contribute to this issue, such as experiencing multiple symptoms including fatigue, and spending approximately three to four hours three times per week or more at the hospital. Previous studies indicated that only a few patients (14.3%) were able to perform normal routine activities, and 4.5% reported that they could only perform minimal activity [14]. Thus, it is important to assist patients and families in coping to changes due to long-term hemodialysis therapy.

Implications to nursing practice

This study has significant implications for practice and future studies. Considering the needs of patients receiving hemodialysis from their own perspectives is highly important to implement patient-centered care approaches and provide high-quality care. Therefore, more attention from nurses and other healthcare providers is needed to assess patient needs individually. In particular, during hemodialysis sessions, nurses are responsible for giving direct care to patients. They should continuously assess patients' needs to ensure patient satisfaction with care and ensure quality of care. In addition, teaching patients about utilizing different coping mechanisms for fatigue is recommended [24]. As it seems from this study, patients only used rest and sleep for self-management of fatigue.

It is recommended that nurses offer greater emotional support to female patients and urge male patients to discuss their psychological requirements and express any negative emotions in a more candid manner. This study can extend nurses' knowledge and enhance their understanding the needs of patients on hemodialysis. Few studies in the literature have explored the needs of patients on hemodialysis, so there is a window for future research in this area, especially in Saudi Arabia. In addition, future research may focus on planning and evaluating intervention plans to fulfill patients’ needs.

Limitations

This qualitative study had a number of limitations. The sample size reflects a small fraction of the number of patients who are undergoing hemodialysis in Saudi Arabia. Moreover, as a consequence of the coronavirus and quarantine lockdown, the research data collection method was changed from planned face-to-face interviews to telephone interviews. In face-to-face interviews, nonverbal communications, such as facial expressions, can be considered. Therefore, replicating this study with face-to-face interviews is a direction for future studies. In addition, the participants were recruited from one city in Saudi Arabia, which may affect the generalizability of the results.

## Conclusions

Hemodialysis therapy affects many aspects of individuals’ lives, which leads to changes in their needs that must be fulfilled. This study highlights the aspects of these needs among hemodialysis patients from their own perspective in Saudi Arabia. Four themes of needs emerged from this study. These factors were related to rest after the hemodialysis session due to fatigue, psychological and emotional needs, changes in role performance, and the need for coping. Consequently, health care professionals must assess patients’ needs frequently to ensure high-quality care.
